# A novel mutant allele of *SSI2* confers a better balance between disease resistance and plant growth inhibition on *Arabidopsis thaliana*

**DOI:** 10.1186/s12870-016-0898-x

**Published:** 2016-09-26

**Authors:** Wei Yang, Ran Dong, Li Liu, Zhubing Hu, Jing Li, Yong Wang, Xinhua Ding, Zhaohui Chu

**Affiliations:** 1 State Key Laboratory of Crop Biology, College of Agronomy, Shandong Provincial Key Laboratory of Agricultural Microbiology, College of Plant Protection, Shandong Agricultural University, Tai’an, 271018 China; 2 College of Life Sciences, Northwest A&F University, Yangling, Shanxi 712100 China; 3 Key Laboratory for Economic Plants and Biotechnology, Kunming Institute of Botany, Chinese Academy of Sciences, Kunming, 650201 China; 4 Present address: Department of Plant Systems Biology, VIB, Ghent University, Technologiepark 927, 9052 Ghent, Belgium

**Keywords:** Auxin, Leaf shape, Plant immunity, Stearoyl-ACP desaturase, Salicylic acid

## Abstract

**Background:**

Resistance and growth are opposing characteristics in plants. *SA INSENSITIVITY OF npr1*-*5* (*SSI2*) encodes a stearoyl-ACP desaturase (S-ACP DES) that has previously been reported to simultaneously enhance resistance and repress growth.

**Results:**

Here, we characterize *ssi2*-*2*, a novel mutant allele of *SSI2* that has two amino acid substitutions. Compared with wild-type and two other mutants of *SSI2*, *ssi2*-*2* showed intermediate phenotypes in growth size, punctate necrosis, resistance to the bacterial pathogen *Pst* DC3000, salicylic acid (SA) content, pathogenesis-related (*PR*) gene levels and 18:1 content. These results indicate that *ssi2*-*2* is a weak mutant of *SSI2*. Additionally, by using *ssi2*-*2* as an intermediate control, a number of differentially expressed genes were identified in transcriptome profiling analysis. These results suggest that constitutive expression of defense-related genes and repression of IAA signaling-associated genes is present in all *SSI2* mutants.

**Conclusions:**

Taken together, our results suggest that the weak *ssi2*-*2* mutant maintains a better balance between plant immunity and vegetative growth than other mutants, consequently providing a basis to genetically engineer disease resistance in crop plants.

**Electronic supplementary material:**

The online version of this article (doi:10.1186/s12870-016-0898-x) contains supplementary material, which is available to authorized users.

## Background

Fatty acids (FAs) are crucial for all living organisms because they are not only a source of energy but are also major components of cellular membranes. Recently, an increasing number of studies has suggested that FAs and their derivatives have important roles as signaling molecules that modulate normal and disease-related processes [1]. In plants, FAs influence a variety of processes in response to both biotic and abiotic stresses [1, 2]. For example, the levels of polyunsaturated FAs in chloroplast membranes affect membrane lipid fluidity, which may affect plant tolerance to temperature stress [3, 4]. In addition, linolenic acid is involved in protein modifications in heat-stressed plants [5]. Azelaic acid, which is derived from C18 FA and contains a double bond at carbon 9, was shown to prime systemic acquired resistance (SAR) [6].

Stearoyl-ACP desaturase (SACPD) is a key enzyme that catalyzes the conversion of stearic acid (18:0) to oleic acid (18:1) during *de novo* FA biosynthesis and produces monounsaturated FAs in plant cells [7, 8]. The *Arabidopsis thaliana* genome has seven SACPD isoforms [8]. *SSI2* is an SACPD isoform that can cause severe growth defects, including spontaneous necrosis and deformed leaves, when mutated. Analysis of plant pathogen resistance showed that the absence of SSI2 activates defense responses and leads to elevated salicylic acid (SA) levels and constitutive expression of pathogenesis-related (*PR*) genes, which results in enhanced resistance to several pathogens, such as *Peronospora parasitica*, *Pseudomonas syringae* pv. *tomato* (*Pst*) and *Cucumber mosaic virus* [9–17]. Others S-ACP-DES isozymes have greatly reduced specific activities compared to SSI2, and knock-out mutations in S-ACP-DES 1 and 4 do not alter defense phenotypes. The observations demonstrate that SSI2 is the predominant SACPD isoform that regulates defense signaling [9].

Using genetic approaches, an *ssi2*-*1* suppressor was isolated, namely *act1*, which encodes a glycerol-3-phosphate (G3P) acyltransferase (ACT1). ACT1 is a key enzyme that catalyzes the acylation of G3P with 18:1 to form lysophophatidic acid (lyso-PA). A mutation in *act1* reduced the conversion of 18:1 to lyso-PA and recovered the content of 18:1 in *ssi2*-*1* mutants, which resulted in growth restoration and reversion of the altered pathogen response in *ssi2*-*1* plants, suggesting that reduced 18:1 might be a direct cause of enhanced resistance and retarded growth [13]. Additionally, restoration of the 18:1 levels can occur via second site mutations in G3P dehydrogenase (*GLY1*) [14] and acyl carrier protein 4 [15].

As chloroplastic 18:1 is distributed in the chloroplast, the effect that 18:1 reduction has on nucleus-encoded resistance genes remains elusive [9, 15, 16]. A possible explanation is that the decreased 18:1 might lead to an accumulation of NOA1 protein, which in turn accelerates NO production and transcriptionally up-regulates NO-responsive nuclear genes, thereby activating disease resistance [18].

This 18:1-derived resistance appears to be conserved among different plant species. Plants with a reduction of SACPD isoforms showed increased resistance to pathogenic bacteria and oomycetes in rice [19] and soybean [20], respectively. Similarly, enhanced resistance to rice blast was observed in rice OsSSI2 knockout mutants [19]. This is not the case in *Arabidopsis*, as the *ssi2*-*1* mutation enhanced resistance to powdery mildew [21] but showed impaired resistance to *B. cinerea* [10]. In addition to pathogen resistance, characterizing an *Arabidopsis fab2* mutant, which is a *SSI2* null mutant that differs from *ssi2*-*1*, revealed that *SSI2* plays a role in plant development and abiotic adaption, particularly to high temperatures. Plants harboring *fab2* mutations are extremely small compared with wild-type plants [22, 23]. Microscopic analysis demonstrated that the *fab2* mutation lead to defective cell expansion in the mesophyll and epidermal layers of leaves. Surprisingly, high temperatures could ameliorate the severe growth defects of *fab2* mutants, which was not correlated with the fatty acid composition. A possible explanation is that this restoration is due to increased membrane fluidity at higher temperature [23].

Defense can be costly to the plant, and the expression of defense genes can have negative effects on plant development, which to some degree counterbalances their positive effects [24]. Lesion mimic mutants (LMM) always have an altered plant form, such as *snc1*-*1*, *lsd2*, *lsd4*, *dll1*, *hrl1*, which display reduced plant size. Further, PCD-induced leaf necrosis may be correlated with the activation of resistance responses [25]. Here, molecular analysis, histological staining and transcriptional profiling reveal that a weak mutation of *SSI2* causes punctate necrotic spots and enhanced resistance to the bacterial pathogen *Pst* DC3000, with a degree of resistance less than that of *ssi2*-*1* and the T-DNA mutant, but the degree of growth disruption was also reduced. This mutant thus provides important information for potential genetic engineering to improve disease resistance.

## Results

### *ssi2*-*2* shows decreased growth and increased disease resistance

**Fig. 1:**
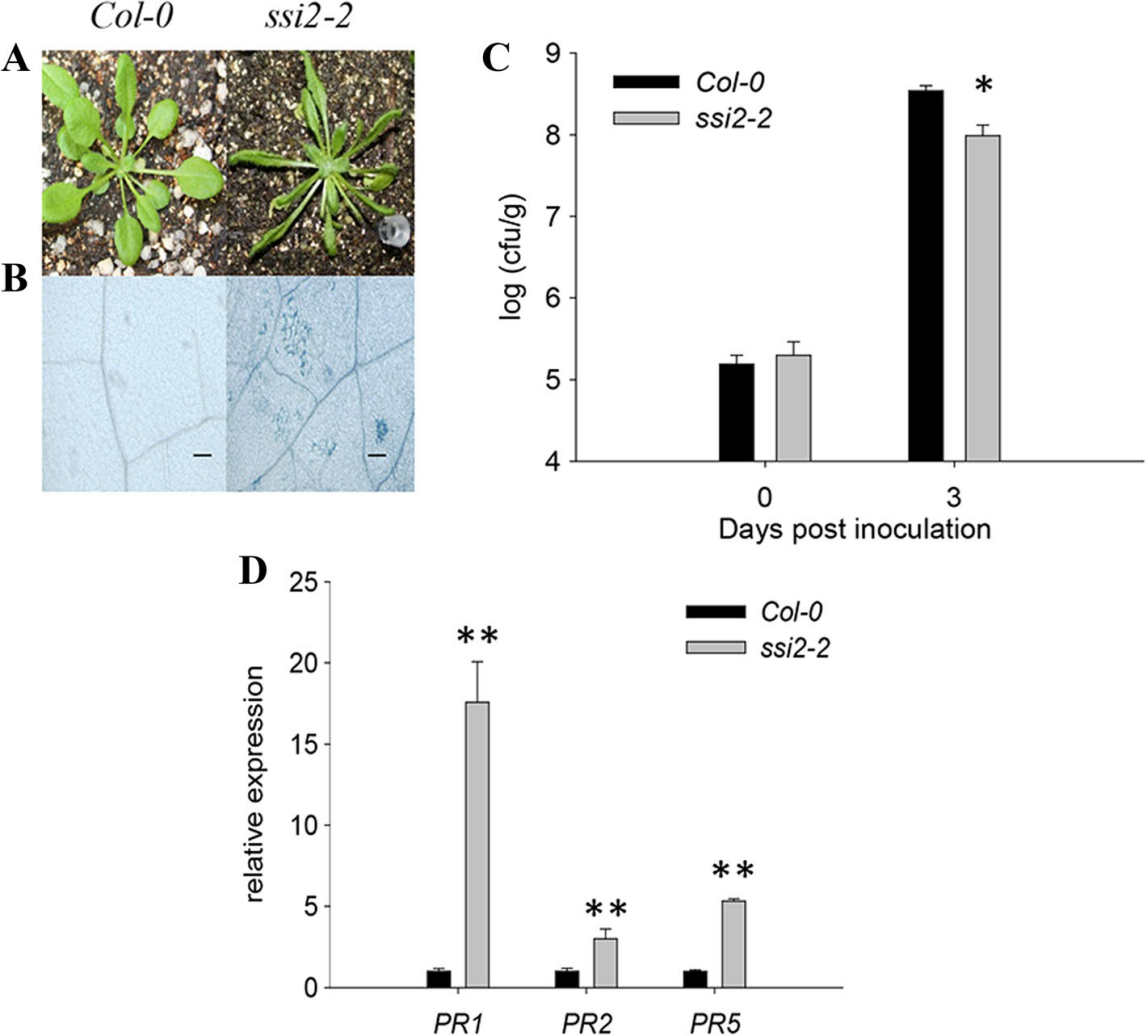
Phenotypic differences between the wild-type and *ssi2*-*2* mutant plants. **a** The phenotype of Col-0 and the *ssi2*-*2* mutant plants grown in a culture chamber at 22 °C for 4 weeks. **b** Col-0 wild-type and *ssi2*-*2* mutants stained with trypan blue. Scale bars = 100 μm. **c** Growth of *Pst* DC3000 in the leaves of wild-type and *ssi2*-*2* mutant plants which cultured for 4 weeks. Three leaves were harvested from infected leaves and then weighed, together homogenized in 10 mM MgCl_2_, diluted 10^5^-or 10^6^-fold, and plated on King’ s B medium. The bacterial population was determined from three replicates at each time point by counting colony-forming units (cfu). The “0 day” point represents 2 h after bacterial inoculation. Each point represents the mean ± standard deviation. The experiments were repeated three times. Significance was determined by Student’s *t* test. * *P* < 0.05. **d** The expression of *PR1*, *PR2*, *PR5* in *ssi2*-*2* mutants. The test performed by quantitative RT-PCR analysis. Transcript abundance of genes was normalized to that of the reference gene ACTIN2 (AT3G18780). The data are shown as means ± SD from three biological replicates. The experiments were repeated three times. ** *P* < 0.01

To dissect the mechanism for plant immunity, we screened for mutants that display significant LMM phenotype using ethyl methanesulfonate (EMS)-mutagenized *Arabidopsis thaliana* ecotype Columbia (Col-0). By close observation, one mutant was found to have many small white spots on its mature leaves and was isolated and designated *ssi2*-*2* based on map-based cloning results (see below). Mature *ssi2*-*2* plants displayed growth defects, as shown by its small and narrow leaves compared to those of wild-type plants (Fig. 1a). To validate whether these white spots were due to programmed cell death (PCD), we carried out trypan blue staining, a widely used approach for selective detection of dead tissues or cells. As expected, many blue spots were found in mature leaves of *ssi2*-*2*, indicating that the white spots result from PCD (Fig. 1b).

To test whether the *ssi2*-*2* mutants have altered pathogen resistance, *Pst* DC3000 was inoculated into WT and *ssi2*-*2* mutant plants. The total number of bacteria in *ssi2*-*2* leaves was significantly lower than that in wild-type leaves at 3 days post-inoculation (dpi), suggesting that *ssi2*-*2* exhibited activated resistance responses (Fig. 1c). The transcriptional levels of several pathogenesis-related genes, including *PR1* (AT2G14610), *PR2* (AT3G57260) and *PR5* (AT1G75040) were significantly up-regulated by 17.6-fold, 3.1-fold and 5.3-fold, respectively, in *ssi2*-*2* compared with wild-type plants (Fig. 1d). These results demonstrate that *ssi2*-*2* is involved in plant disease resistance.

### Cloning of *ssi2*-*2* showed two nucleotide substitutions in the *SSI2* coding sequence

**Fig. 2:**
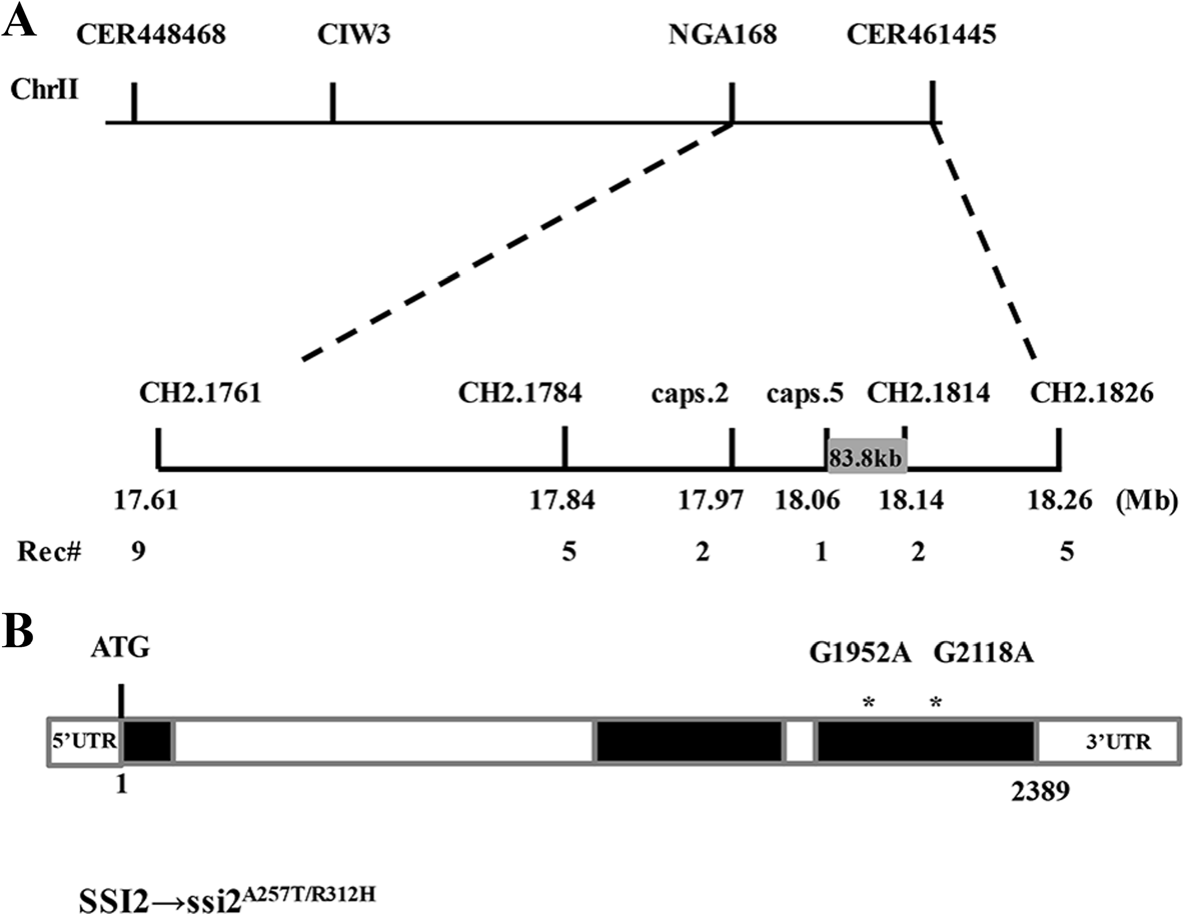
The *ssi2*-*2* mutant harbors a new allele of *SSI2*. **a** Physical mapping of *ssi2*-*2*. Rough mapping showed that the *ssi2*-*2* gene is located between markers NGA168 and CER461445 on chromosome 2 of *Arabidopsis*. Fine mapping revealed the gene between the primers of CAPS5 and CH2.1814, a range of 83.8 kb. The numbers below the molecular markers indicate the recombinant events detected between the *ssi2*-*2* locus and the marker. **b** Structure of the *ssi2*-*2* gene, AT2G43710. AT2G43710 encodes stearoyl desaturase and has three exons and two introns; sequence analysis revealed two point mutations in the third exon of the gene, namely G1952A and G2118A, resulting in changes in amino acids 257 and 312

To clone the gene, a segregating F_2_ population with approximately 6,000 individuals was generated. In this process, we found that all F_1_ plants showed WT morphology, and all F_2_ seedlings exhibited a near 3:1 (134:43) segregation of normal:narrow (WT:*ssi2*-*2*) leaf phenotypes, indicating that *ssi2*-*2* is caused by a single-gene recessive mutation. Through rough mapping, the mutated gene was located on chromosome II in a 3.4 Mb region between NGA168 and CER461445. To facilitate fine mapping, new SSLPs and CAPS markers were developed in this region and 20 new polymorphic markers were generated. By using these newly developed markers, the mutated gene was finely mapped to an 83.8 kb region (Fig. 2a). We then designed 62 pairs of sequencing primers to re-sequence the entire candidate region. Compared to the reference sequence data of Col-0, two separate point substitutions were found in the coding region of *SSI2* (AT2G43710), which led to amino acid alterations of A257T and R312H (Fig. 2b).

**Fig. 3:**
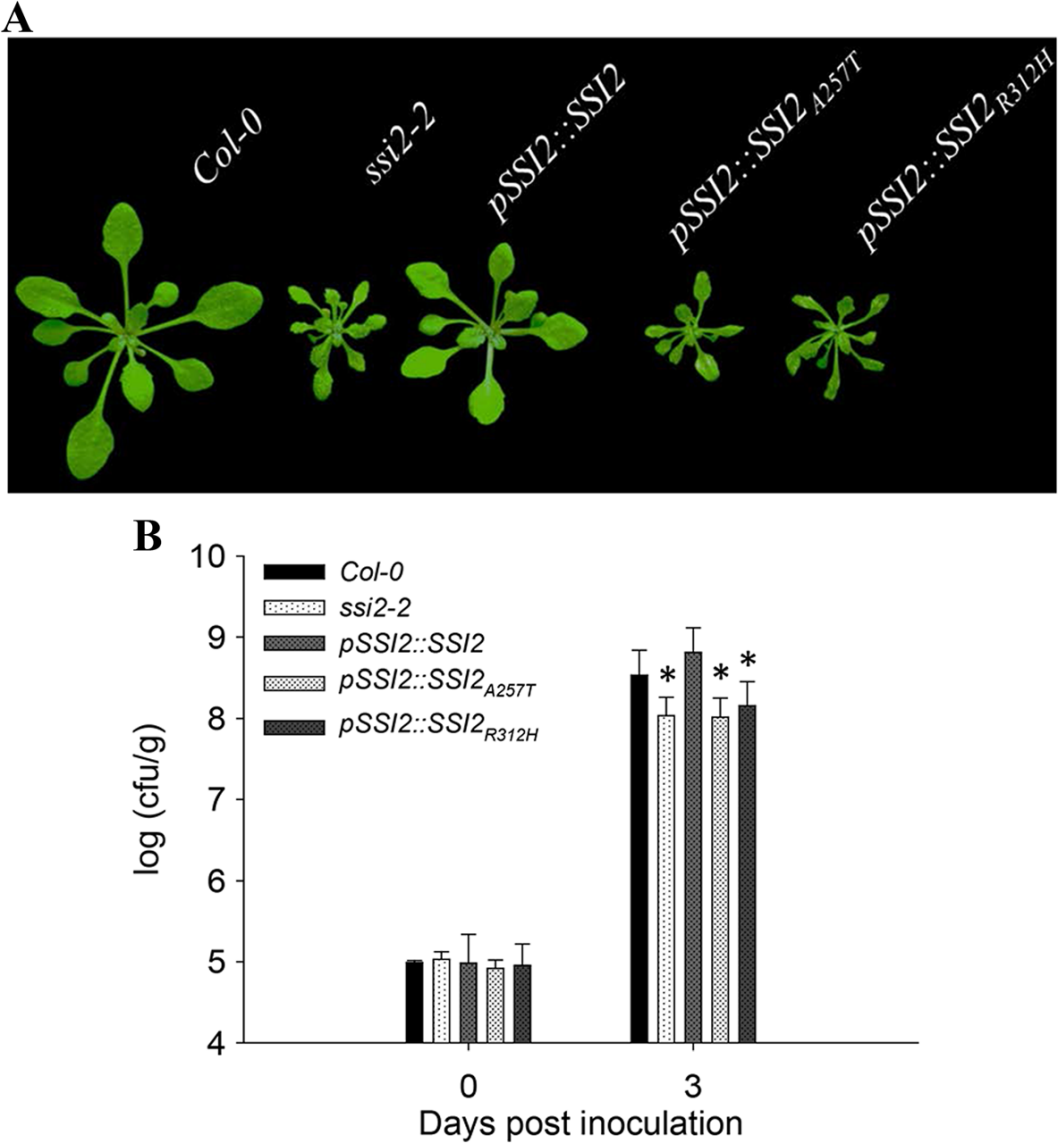
Complementation of the *ssi2*-*2* mutant. **a** Phenotypes of the transgenic complementation lines. Constructs harboring each single mutation did not rescue the dwarf phenotype of *ssi2*-*2*. Plants were photographed 3 weeks after germination. **b** The bacterial population of *Pst* DC3000 in the leaves of wild-type Col-0, *ssi2*-*2* mutant and transgenic complementation lines. The seedlings were cultured for 4 weeks. Three leaves were harvested from infected leaves and then weighed, together homogenized in 10 mM MgCl_2_, diluted 10^5^-or 10^6^-fold, and plated on King’ s B medium. The bacterial population was determined from three replicates at each time point by counting colony-forming units (cfu). The “0 day” point represents 2 h after bacterial inoculation. The data are shown as means ± SD from three biological replicates. The experiments were repeated three times. Significance was determined by Student’s *t* test. * *P* < 0.05

To establish a direct causal link with these mutations, an intact *SSI2* genomic DNA fragment driven by the native *SSI2* promoter was introduced into *ssi2*-*2* plants. Normal growth size was observed in *pSSI2*::*SSI2* transgenic offspring (Fig. 3a), confirming that growth defects of *ssi2*-*2* are caused by *SSI2* mutations. Because *ssi2*-*2* has two mutations at the SSI2 locus, two different mutated *SSI2* fragments, *pSSI2*::*SSI2*_*A257T*_ and *pSSI2*::*SSI2*_*R312H*_ (Additional file 1: Figure S1c), were also transformed into *ssi2*-*2* mutants. Surprisingly, both constructs failed to complement the developmental and resistance phenotypes (Fig. 3a, b), demonstrating that both mutated amino acids in *ssi2*-*2* are key for the functional SSI2 phenotype.

*SSI2* encodes a stearoyl-ACP desaturase (S-ACP-DES) that catalyzes the production of oleoyl-ACP (18:1-ACP) from stearoyl-ACP (18:0-ACP) [10]. The previously characterized recessive mutant *ssi2*-*1* lacks nearly 90 % of this enzyme activity and exhibits pleiotropic phenotypes [10]. The *ssi2*-*1* mutant was originally identified as a genetic suppressor of *npr1*-*5* and exhibits constitutively activated plant defense responses without pathogen infection [26]. Based on these previous results and our current analyses, our newly isolated allele was renamed *ssi2*-*2*.

### *ssi2*-*2* shows intermediate phenotypes in development and disease resistance

**Fig. 4:**
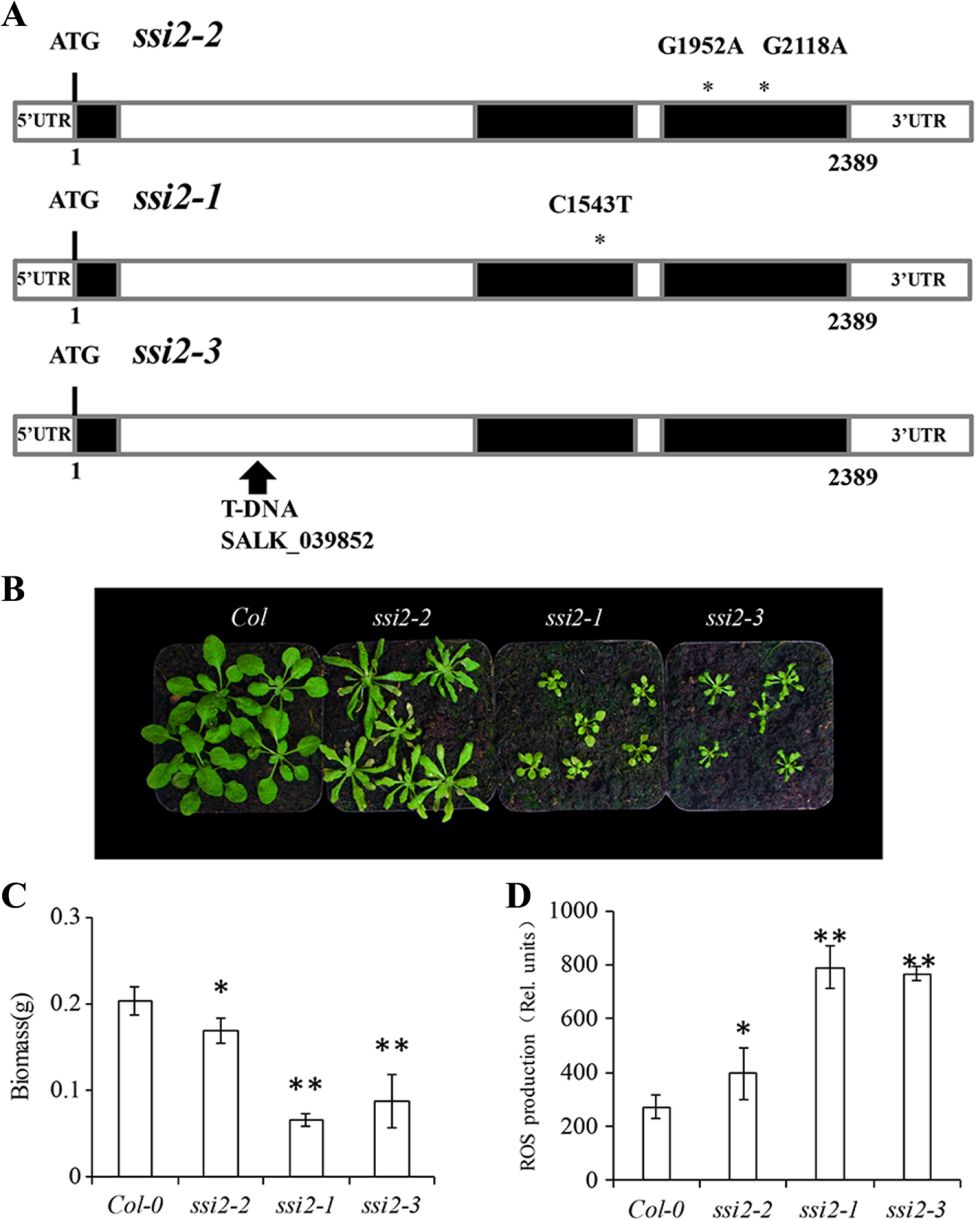
Allele structure and phenotypic differences in *ssi2*-*1*, *ssi2*-*2* and *ssi2*-*3*. **a** The C-to-T mutation in *ssi2*-*1* at nucleotide position 1543 changes the leucine (L) at amino acid position 146 to a phenylalanine (F) [10]. The TAIR database shows the T-DNA insertion in the first intron of *SSI2* in *ssi2*-*3* (SALK_039852). **b** The phenotype of the Col-0 wild-type and different *ssi2* lines grown in a culture chamber at 22 °C for 4 weeks. **c** Biomass analyses of the indicated lines. The wild-type and mutants were cultured for 4 weeks at 22 °C. Data were analyzed by one-way ANOVA, and mean separation was calculated by multiple comparison Tukey’s test (*P* < 0.05). Nine plants were used as biological replicates for each. **d** ROS quantitation in the leaves of wt (Col-0), *ssi2*-*1*, *ssi2*-*2* and *ssi2*-*3* plants. The leaves directly treated with 10 mM H_2_DCF-DA dissolved in PBS at 37 °C for 30 min. The epidermis was isolated and the fluorescence intensity was monitored. Quantification of ROS was performed using an ImagePro Plus analysis package

To further study the function of *SSI2*, we analyzed *ssi2*-*1* mutants [10] and a T-DNA insertion line (named *ssi2*-*3*) (Additional file 1: Figure S1a, b). Based on the sequence information in the TAIR database, the T-DNA insertion of *ssi2*-*3* is located in the first intron of *SSI2* (Fig. 4a). Obvious differences in phenotype were observed after cultivating these mutants in the growth chamber. The biomasses of *ssi2*-*1* and *ssi2*-*3* plants were 38.13 and 51.57 % compared to that of *ssi2*-*2*, respectively (Fig. 4b, c). Trypan blue staining showed that all three *ssi2* mutants exhibited clear cell necrosis; differences in the necrotic areas were not noticeable among the three mutants (Additional file 1: Figure S2).

ROS burst is an important symbol of the plant disease resistance. Quantitation of ROS showed that the *ssi2*-*2* mutant accumulated lower ROS level than the *ssi2*-*1* and *ssi2*-*3* mutants (Fig. 4d). Nitroblue tetrazolium (NBT) and 3, 3′-diaminobenzidine (DAB) staining detected accumulation of superoxide and peroxide, respectively. The staining patterns confirmed the model of ROS quantitation (Additional file 1: Figure S2). We used aniline blue staining to monitor callose deposition in leaf tissues. Bright blue staining in the mutants indicated that there was spontaneous callose deposition in the leaves of all three mutant lines (Additional file 1: Figure S2). These results are consistent with the known association between SSI2 and pathogen resistance.

**Fig. 5:**
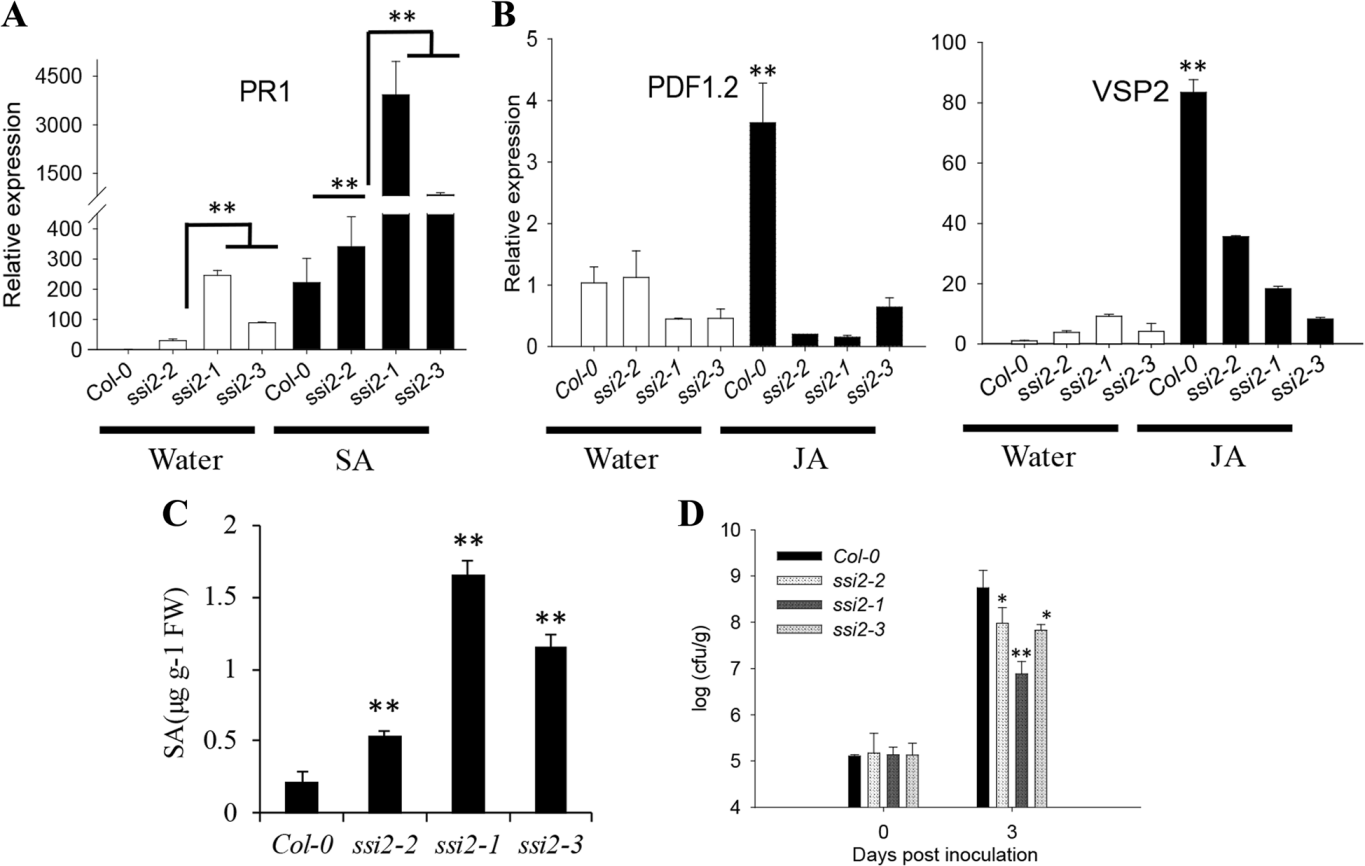
Marker gene expression, SA content and bacterial growth in Col-0 and different *ssi2* mutant lines. Col-0 and different *ssi2* mutant lines were treated with water and 50 μM salicylic acid (SA) or 50 μM jasmonic acid (JA), and samples were taken 24 h after treatment. The expression levels of *PR1* (**a**) and *PDF1.2*, *VSP2* (**b**) are shown. The test performed by quantitative RT-PCR analysis. Transcript abundance of genes was normalized to that of the reference gene ACTIN2 (AT3G18780). The data are shown as means ± SD from three biological replicates. The experiments were repeated three times. ** *P* < 0.01. **c** SA levels in the leaves of wt (Col-0), *ssi2*-*1*, *ssi2*-*2* and *ssi2*-*3* plants. **d** Growth of *Pst* DC3000 in the leaves of wild-type and different *ssi2* mutant lines cultured for 4 weeks. The 0 d time point represents 2 h after bacterial inoculation. The data are shown as means ± SD from three biological replicates. The experiments were repeated three times. Significance was determined by Student’s *t* test. *Indicates significant differences at *P* < 0.05, ** Indicates significant differences at *P* < 0.01

In wild-type *Arabidopsis* plants, *PR* genes can be activated by various types of biotic stress. *PR1* is a marker gene for the SA signaling pathway in plants [27]. The *ssi2*-*1*, *ssi2*-*2* and *ssi2*-*3* mutants were capable of self-activating *PR1* expression, which showed increases of 245.5-, 29.5- and 88.9-fold, respectively, compared to the wild-type plants in the absence of exogenous SA, respectively, suggesting that *SSI2* deficiency leads to constitutive activation of SA signaling. After treatment with exogenous SA, the expression of wild-type plants increased by 221.8-fold, while the *ssi2*-*1*, *ssi2*-*2* and *ssi2*-*3* plant expression levels increased by 3926.8-, 340.6- and 837.6-fold (Fig. 5a), respectively, indicating that *PR1* expression can be further up-regulated after SA treatment.

*PDF1.2* and *VSP2* can serve as marker genes for JA signaling [28]. The expression of *PDF1.2* in *ssi2*-*2* was similar to that of wild-type, but in *ssi2*-*1* and *ssi2*-*3* mutants, the expression of *PDF1.2* was significantly reduced to 0.45- and 0.46-fold, respectively, of that of the wild-type. After treatment with exogenous JA, the expression of wild-type plants was up-regulated by approximately 4-fold, while that of the *ssi2*-*1*, *ssi2*-*2* and *ssi2*-*3* mutants increased by approximately 0.3-fold compared to the wild-type. A similar result also observed in *VSP2* gene expression analysis (Fig. 5b). These results are consistent with the northern blot results showing that several JA-inducible defense responses are impaired in *ssi2*-*1* plants [10] and demonstrating that the JA signaling pathway is also impaired in *ssi2*-*2* and *ssi2*-*3*.

We also measured the SA content in mutant leaves. As shown in Fig. 5c, *ssi2*-*1*, *ssi2*-*2* and *ssi2*-*3* mutants had approximately 8-, 2- and 5-fold higher levels of SA than wild-type plants, respectively. Consistent with these results, all *ssi2* mutants exhibited resistance phenotypes. The total amount of bacteria in wild-type leaves was 180.8-, 14.7- and 20.9-fold higher than that in *ssi2*-*1*, *ssi2*-*2* and *ssi2*-*3* leaves (Fig. 5d). In terms of resistance, the *ssi2*-*1* mutant showed the strongest resistance, while the *ssi2*-*2* mutant had the weakest resistance.

*SSI2* is located in the chloroplast. We also determined the subcellular location of *SSI2* in the mutant *ssi2*-*2* and *SSI2*_*A257T*_, *SSI2*_*R312H*_ mutants. Similar to the WT, a protein with two mutated amino acids (*ssi2*-*2*) or single amino acid mutations (*SSI2*_*A257T*_, *SSI2*_*R312H*_) was located in the chloroplast (Additional file 1: Figure S3), indicating that point mutations in *ssi2*-*2* did not affect the subcellular localization of the protein.

### The 18:1 content is decreased in *ssi2*-*2*

**Fig. 6:**
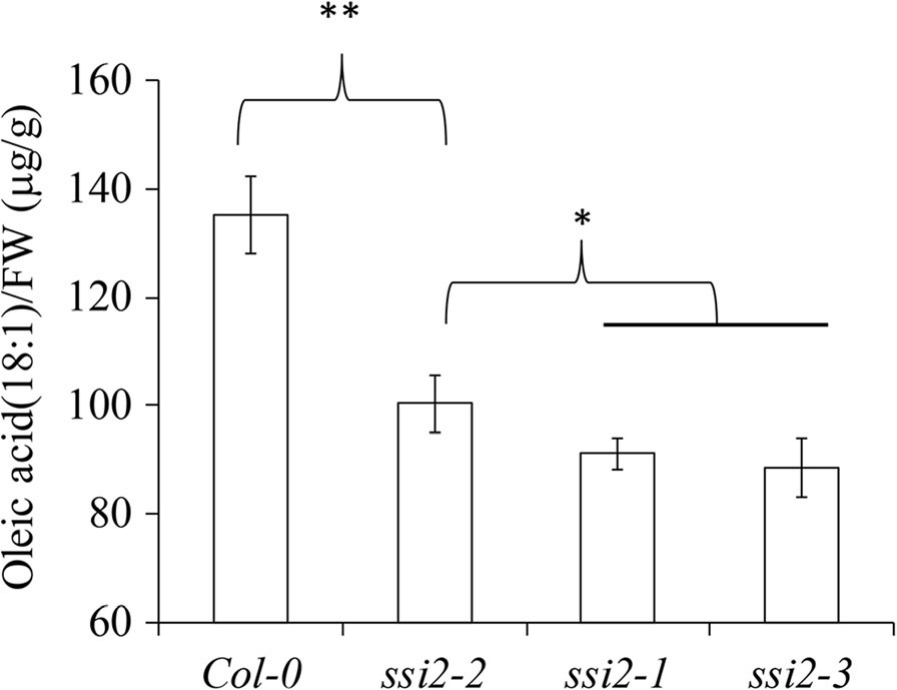
Oleic acid content in leaf tissues of Col-0, *ssi2*-*1*, *ssi2*-*2* and *ssi2*-*3* plants. All measurements were made on plants grown at 22 °C for 4 weeks, and data are presented as μg ± SD (*n* = 6). Significance was determined by Student’s *t* test. *Indicates significant differences at *P* < 0.05, ** Indicates significant differences at *P* < 0.01

Because *SSI2* is an isoform of S-ACP-DES that converts 18:0 to 18:1 [10], the *ssi2*-*2* mutation may alter its activity, which in turn would affect 18:1 production. To examine this, we directly measured the oleic acid content of WT and *ssi2* mutants. In wild-type plants, the oleic acid content was 135.35 ± 7.13 μg/g. In contrast, a significant decrease was observed in all *ssi2* mutants (91.2 ± 3 μg/g for *ssi2*-*1*, 100.37 ± 5.41 μg/g for *ssi2*-*2* and 88.71 ± 5.43 μg/g for *ssi2*-*3*). Compared with *ssi2*-*1* and *ssi2*-*3* mutants, the *ssi2*-*2* mutants had higher levels of oleic acid (Fig. 6), indicating that *ssi2*-*2* is a weak allele.

### SSI2 regulated the expression of genes involved in SA and IAA pathways

**Fig. 7:**
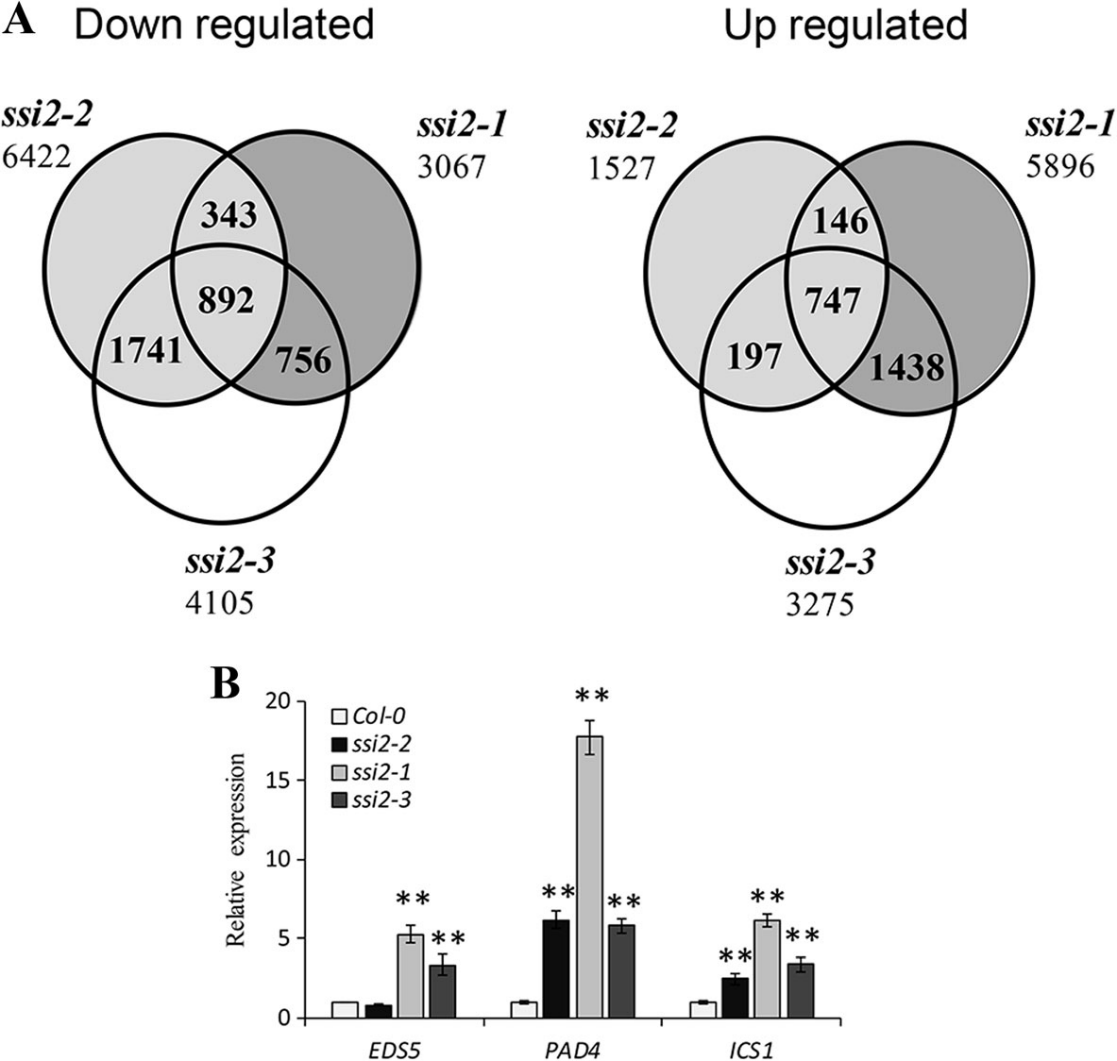
Gene expression profiling. **a** Statistics for transcriptome sequencing of Col-0 and three *ssi2* mutants. **b** Quantitative RT-PCR analysis of three SA pathway genes to validate the RNA-seq data of up-regulated genes. The data are shown as means ± SD from three biological replicates. The experiments were repeated two times. Significance was determined by Student’s *t* test. ** Indicates significant differences at *P* < 0.01

To elucidate the mechanism underlying the observed phenotype of *ssi2* mutants, an RNA sequencing experiment was conducted (Additional file 2: Table S1). Compared to wild-type, thousands of genes displayed significant changes in transcript levels in all mutants. Specifically, the *ssi2*-*2* mutant had 1,527 up-regulated genes and 6,422 down-regulated genes. The total number of up-regulated genes (5,896 and 3,275) and down-regulated genes (3,067 and 4,105) was identified in *ssi2*-*1* and *ssi2*-*3* mutants, respectively (Fig. 7a).

Because all three mutants showed increased resistance to *Pst* DC3000 and reduced growth size, after integrated analysis of the RNA-seq data, the common 747 up-regulated genes and 892 down-regulated genes were identified in all three mutants. Using the *ssi2*-*2* mutant as an intermediate control, 484 of the 747 up-regulated genes showed reduced expression in the *ssi2*-*2* mutant compared to the *ssi2*-*1* and *ssi2*-*3* mutants, in accordance with the “weak mutant” phenotype. GO annotations were assigned to the differentially expressed genes in the mutant lines, and PANTHER [29] analysis revealed a significant enrichment in immune system processes and SA signaling pathways among these genes (Additional file 2: Table S2), which is consistent with the previous conclusion that the SA pathway is associated with low levels of oleic acid. To validate the data of the RNA-seq experiments, the expression levels of *PAD4* (AT3G52430), *EDS5* (AT4G39030), and *ICS1* (AT1G74710) were tested by quantitative RT-PCR and exhibited the same expression pattern (Fig. 7b). In addition, genes categorized as response to chitin (GO:0010200) and response to nitrogen compounds (GO:1901698) were significantly enriched in the analysis, implying that *SSI2* also mediated additional resistance responses.

**Fig. 8:**
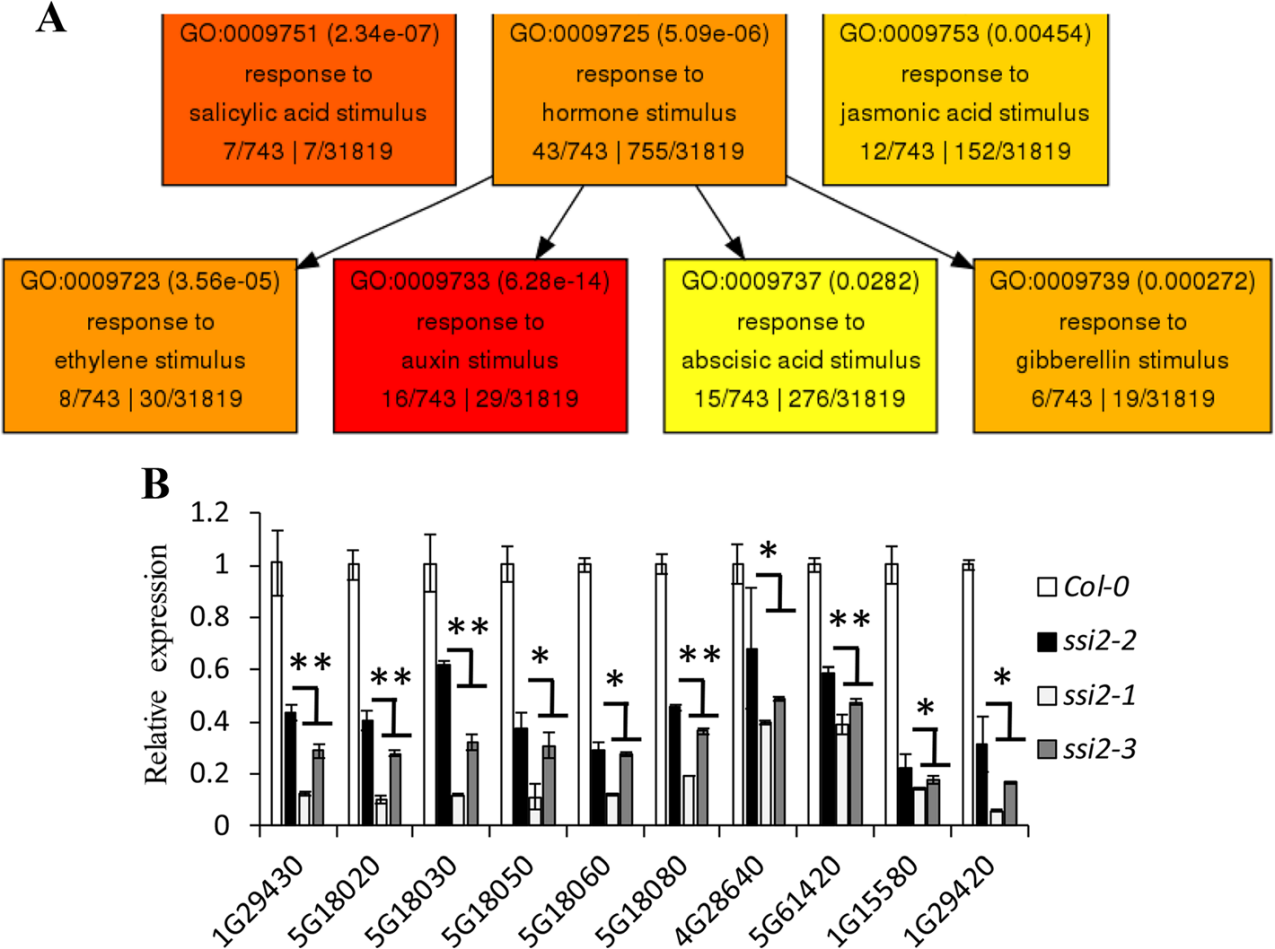
Several IAA pathway genes were significant down-regulated in SSI2 mutant. **a** The description of agriGO analysis about the hormones-related GO enrichment. The numbers marked in parentheses were *P* values. The grid marked with deeper color-coded means higher significantly enrichment. **b** Quantitative RT-PCR analysis of ten IAA pathway genes to validate the RNA-seq data of down-regulated genes. The data are shown as means ± SD from three biological replicates. The experiments were repeated two times. Significance was determined by Student’s *t* test. *Indicates significant differences at *P* < 0.05, ** Indicates significant differences at *P* < 0.01

We tried to analyze the molecular mechanism controlling the developmental phenotype of the *ssi2* mutants. Abnormal growth phenotypes are often closely related to plant hormones. By agriGO [30] analysis, for the hormone-related GO annotation, the highest degree of enrichment of the 892 down-regulated genes was response to auxin stimulus (GO:0009733) (Fig. 8a), suggesting that reduced oleic acid may inhibit auxin-mediated pathways, affecting the developmental process. Further analysis identified that 24 genes out of 892 genes were auxin-related (Additional file 2: Table S3). By using quantitative RT-PCR, 10 out of 24 auxin-related genes showed less repression in the *ssi2*-*2* mutant than in the *ssi2*-*1* and the *ssi2*-*3* mutants. Notably, *SAUR20* (AT5G18020), *SAUR21* (AT5G18030), *SAUR22* (AT5G18050), *SAUR23* (AT5G18060), and *SAUR24* (AT5G18080) belong to a subgroup of SMALL AUXIN UP RNA (SAUR) genes which have been reported to be connected with plant leave size [31]. Another two SAUR proteins, *SAUR61* (AT1G29420) and *SAUR62* (AT1G29430), play a role in organ development [32]. IAA5 (AT1G15580) and *IAA11* (AT4G28640) as two early auxin-induced transcription factors, were also significantly repressed in SSI2 mutants. These results imply that the auxin mediated pathway combines with the SSI2-mediated pathway to produce the narrow-leaf dwarf phenotype.

## Discussion

The *ssi2*-*1* mutant was previously identified as being resistant to pathogens, and the *ssi2*-*1 npr1* double mutant was susceptible to *Pst* DC3000. It was suggested that resistance to *Pst* DC3000 which was mediated by *ssi2*-*1*, depended on *NPR1*. However, depletion of *NPR1* could not suppress the increased SA content, development of spontaneous necrotic spots and constitutively high expression of *PR* genes in *ssi2*-*1*, demonstrating that *SSI2* also mediates a NPR1-independent pathway [26]. The *ssi2*-*2* mutant also displayed constitutive expression of *PR* genes (Fig. 1d, Additional file 2: Table S2), spontaneous necrosis and pathogen resistance, demonstrating that *ssi2*-*2* is a novel allele of *SSI2*. Applying exogenous SA to *ssi2* mutants increased the expression of *PR* genes. These results, combined with the fact that *SSI2* mutants are only resistant to biotrophic pathogens and are highly susceptible to necrotrophic pathogens, suggest that SSI2-mediated resistance is principally dependent on the SA pathway. Mutations in *SSI2* may regulate the SA signaling pathway, but are not necessarily involved in this pathway, and *SSI2* functions upstream of the *EDS1* gene [12].

Compared to the *ssi2*-*1* and *ssi2*-*3* mutants, the overall growth of *ssi2*-*2* plants was significantly higher (Fig. 4b, c). *ssi2*-*2* carries two amino acid substitutions that are not located in the central activated region of the enzyme. The improved growth might be explained by higher enzymatic activity in *ssi2*-*2* plants compared to *ssi2*-*1* and *ssi2*-*3* plants which is supported by a smaller decrease in oleic acid in *ssi2*-*2* plants (Fig. 6). Several indicators of disease resistance, such as the leaf ROS levels, *PR* gene expression levels and number of bacterial colonies after infection, were also intermediate in the *ssi2*-*2* mutant, between those of wild-type plants and *ssi2*-*1* and *ssi2*-*3* mutants (Figs. 4 and 5). These characteristics support the conclusion that *ssi2*-*2* is a new weak mutant allele of *SSI2*. By sequence alignment, the corresponding site of *Arabidopsis* Ala^257^ is Ala^220^ in castor, which is close to the pairs of iron binding helices [33]. Iron binding plays a key role in interrupting the C-H bond of the fatty acid chain [34]. Does the A257T mutation in *Arabidopsis* interfere with iron binding, thereby reducing enzyme activity? It remains unclear. Via subunit structure analysis in castor, Arg^274^, which corresponds with Arg^312^ in *Arabidopsis*, is predicted to interact with Asp^358^ after the eighth α-helix and before the second β-hairpin [33]. Therefore, the R312H mutation in *ssi2*-*2* was hypothesized to interfere with the structure of SSI2, resulting in decreased enzyme activity.

Decreased enzymatic activity alone seems insufficient to explain SSI2-mediated resistance. The 18:1 content was similar between *ssi2*-*1* and *ssi2*-*3* plants, but the SA content in *ssi2*-*1* leaves was much higher than in *ssi2*-*3* leaves, and the resistance of the *ssi2*-*1* mutant was also obviously stronger than that of the *ssi2*-*3* mutant. One explanation is that SSI2 interacts with other proteins or macromolecules, that are necessary for SSI2-mediated signaling.

What is the relationship between reduced oleic acid levels and disease resistance? In expression profiling analysis, we focused on two specific enriched GO items (response to chitin GO:0010200 and response to nitrogen compound GO:1901698), considering the facts that 18:1 regulates NO production in the chloroplast [18] and chitin is regarded to be a typical pathogen-associated molecular patterns (PAMP) [35]. Lower 18:1 levels perhaps not only regulated the downstream SA signaling pathway but also acted as an earlier signal in the plant resistance response.

We also observed that many auxin-related genes showed altered transcriptional levels in the mutants (Additional file 2: Table S3). Specifically, some *SAUR* and *IAA* genes were down-regulated and confirmed by qPCR (Fig. 8b), suggesting that these IAA response genes are probably involved in the regulation of leaf development in SSI2 mutants. Generally, elevated salicylic acid inhibits pathogen growth by through repression of the auxin signaling pathway [36]. Because SSI2 mutants activated SA and other disease resistance signaling pathways, these pathways may have antagonized the IAA signaling pathways and regulated SAUR-mediated developmental signaling by an unknown mechanism.

## Conclusions

Previous studies have demonstrated the genes involved in photosynthesis and growth were down-regulated during induced resistance [37]. However, few studies have focused on the costs and trade-offs associated with induced resistance to pathogens. The limiting effect of disease resistance on yield should continue to be studied. The *ssi2*-*1* and *ssi2*-*3* mutants show dwarf phenotypes, and SACPD gene silencing significantly reduced soybean plant height and seed yield [20]. These results demonstrate that high disease resistance often comes at a great cost to plants. Accordingly, the weak *ssi2*-*2* mutant presented a better balance between resistance and growth which could be a great advantage in crop breeding, especially in plants with lower fatty acid requirements, such as vegetables and trees, in which pathogen resistance could be gained with little effect on growth. For crops with stricter requirements in terms of fatty acid composition, such as canola and corn, we could also develop a strategy to breed lines that have leaves that are low in oleic acid, but that have seeds that contain an unchanged fatty acid composition to achieve a balance between plant growth and pathogen resistance. Our research therefore provides a theoretical basis for choosing effective resistance breeding strategies.

## Methods

### Plant cultivation

*ssi2*-*1* mutant seeds were kindly provided by Prof. Kachroo, and SALK_039852 (*ssi2*-*3*) seeds were obtained from The Arabidopsis Information Resource (http://www.arabidopsis.org). Seeds of the *Arabidopsis* Col-0 and Ler-0 ecotypes and other mutant lines were first surface-sterilized with 5 % (v/v) sodium hypochlorite and 75 % (v/v) ethanol and then thoroughly washed six times with sterile water. After vernalization at 4 °C for 2 d in darkness, *Arabidopsis* seeds were grown in soil or on 1/2 Murashige and Skoog (MS) medium containing 1 % (w/v) sucrose and cultured in a growth chamber. The growth chamber was controlled at an irradiance of 120 μmol quanta m^−2^ sec^−1^ at 22 °C with 85 % relative humidity under 12 h light and 12 h dark cycles. A nutrient solution was supplied with water every 3 days to sustain plant growth.

### Mutant screen and map-based cloning

Approximately 30,000 M_2_ plants were screened at 22 °C for lesion mimic phenotypes. To isolate the *ssi2*-*2*, a homozygous mutant plant was first crossed with Ler-0 to generate F_1_ progeny, which in turn were self-pollinated to produce F_2_ progeny. Bulked segregation analysis was performed on pools of 20 plants with simple sequence length polymorphisms (SSLPs) by PCR amplification, and the 1,200 narrow-leaf plants were used for genetic mapping by PCR amplification of SSLPs. SSLPs and derived cleaved amplified polymorphic sequence (CAPS) markers between the Col-0 and Ler-0 ecotypes were used for fine mapping. Primers were designed with (http://helix.wustl.edu/dcaps/dcaps.html). The primers used in map-based cloning are listed in Additional file 2: Table S4.

### Generation of transgenic plants

For the *pSSI2*::*SSI2* transgenic line, a 4.2-kb genomic fragment containing the *SSI2* promoter region and coding sequence was amplified by PCR from the wild-type (Col-0) and inserted into the pCXGFP-P [38] vector by TA cloning. We generated the single mutation AtSSI2 transgenic construct with PCR-based mutation using the Fast Mutagenesis System (FM111-01, Transgen Biotech, Beijing, CN). The primers for vector construction are listed in Additional file 2: Table S5. The binary vector was transformed into *ssi2*-*2* plants using the floral dip method [39]. Transgenic plants were selected on plates containing hygromycin. The complementation test was confirmed by genotypic analysis of the T_2_ plants.

### Quantitative RT-PCR analysis

Total RNA was isolated from 100 mg plant tissue with TRI reagent according to the manufacturer’s instructions (T9424, Sigma-Aldrich, USA). RNA (0.5 μg) was used for first-strand cDNA synthesis with a PrimeScript^TM^ RT reagent kit with gDNA Eraser (TaKaRa, Dalian, CN). Quantitative PCR was performed with SYBR® Premix Ex Taq™ (Tli RNaseH Plus) on an IQ5 Real-Time PCR System (Bio-Rad, USA). The PCR was performed as previously described [40]. *AtACTIN2* of *Arabidopsis* was used as an internal control to standardize the results. For each gene, qRT-PCR assays were repeated at least twice with triplicate runs. The relative expression levels were determined using the 2^-⊿⊿Ct^ analysis method. The sequences of the primers for all of the detected genes are listed in Additional file 2: Table S5.

### Plant disease resistance assay

For disease resistance assays, 28-d-old plant leaves were sprayed with virulent *Pseudomonas syringae* pv. *tomato DC3000* at optical densities at 600 nm of 0.2. Bacterial cultures were grown overnight in King’s B medium containing rifampicin and/or kanamycin. Inoculation with 10 mM MgCl_2_ was used as a mock treatment. Inoculated plants were covered with a clear plastic dome to maintain humidity throughout the course of the experiment. At 0 and 3 dpi, the treated leaves were harvested. The leaves were homogenized in 10 mM MgCl_2_, diluted 10^3^- or 10^4^-fold, and plated on King’s B medium. *P. syringae*-related experiments were repeated three times for every genotype analyzed.

### DAB, NBT, trypan blue and aniline blue staining

DAB staining and NBT staining were performed as previously described [41]. Briefly, the seedlings were immersed in DAB solution (1 mg mL^−1^) and NBT solution (1 mg mL^−1^) at room temperature for 8 h. The stained seedlings were then transferred to 70 % (v/v) ethanol to remove chlorophyll and visualize brown and blue spots, which represented H_2_O_2_ and O_2_^−^, respectively. Leaves of 28-d-old plants were stained in a lactophenol trypan blue solution as described previously [42]. Ethanol (70 % v/v) was used to remove the chlorophyll, and leaves were then photographed. Callose depositions were visualized using aniline blue (0.01 % in 150 mM KH_2_PO_4_, pH 9.5) as described previously [26]. Stained leaves were stored in a 50 % glycerol solution in the dark and subsequently examined for fluorescence using a Nikon 90I microscope with a standard filter block for ultraviolet fluorescence UV-2A (excitation 330–380 nm).

### ROS quantitation

The ROS level was determined using 2, 7-dichlorodihydro-fluorescein diacetate (H_2_DCF-DA) (Beyotime Institute of Biotechnology, Haimen, China). The leaves were directly treated with 10 mM H_2_DCF-DA dissolved in PBS at 37 °C for 30 min. The epidermis was isolated and the fluorescence intensity was monitored with an excitation wavelength at 488 nm and emission wavelength at 525 nm. Quantification of ROS was performed using ImagePro Plus software as previous study [43].

### Leaf fatty acid (FA) analyses and SA quantification

FA analysis was performed as previously described [16]. Briefly, FA extraction was carried out by placing leaf tissue in 2 mL of 3 % sulfuric acid in methanol. After a 30 min incubation at 80 °C, 1 mL of hexane with 0.001 % butylated hydroxytoluene was added. The hexane phase was then transferred to vials for gas chromatography (GC). The SA concentration was quantified by GC-MS analysis according to Brader *et al*. [44].

### Fluorescence microscopy analysis

GFP was used as a reporter to investigate the subcellular localization of different gene allele fragments *in planta*. The full-length SSI2 gene and three mutation fragments were cloned into vector pEarley103 (Invitrogen, CA, USA). The constructs were introduced into the *A. tumefaciens* strain GV3101 and transiently expressed in the tobacco epidermis. Live plant imaging was performed on a Zeiss LSM510 META confocal microscope (Carl Zeiss) using a 40× C-Apochromat water immersion objective lens.

### Transcriptional profiling

Total RNA was isolated from 4-week-old plants using TRI reagent. RNA was used to create a cDNA library for sequencing on an Illumina HiSeq™ 2000. RNA sequencing was performed with the commercial service from Chinese National Human Genome Center at Shanghai (CHGC, www.hanyubio.com). The DEGseq package with MARS (MA plot-based method with random sampling model) [45] was used to analyze the data. A sequence data set comprising *Arabidopsis* Col-0 unigenes from the TAIR database (http://www.arabidopsis.org) was used as the reference gene set for read mapping. GO term annotations and WEGO were used to classify their putative functions. Differences in gene expression were calculated for each contig in every sample-pair. FDR values of less than 0.001 and a signal strength log_2_ ratio ≥ 1 or ≤ −1, i.e., 2-fold increase or decrease, respectively, were used as the cut-offs for significant differences.

### Data treatment

The biomass data were analyzed by one-way ANOVA followed by the Tukey test. Quantitative data were analyzed by Student’s *t* test (two-tail *t* test with equal variances; Microsoft Excel) to determine the significant differences between wild-type and mutant plants. *P* < 0.05 is indicated by an asterisk, and *P* < 0.01 is indicated by two asterisks.

## Additional files

Additional file 1: Figure S1. Mutant test and transgenic plant confirmation. **Figure S2.** Cytological staining of Col-0 and different ssi2 mutant lines. **Figure S3.** Quantitative RT-PCR analysis of 14 auxin related genes. **Figure S4.** Subcellular localization of SSI2 and mutant fragments. (PPTX 1923 kb) Additional file 2: Table S1. EXCEL file with RNA-seq data. **Table S2.** GO analysis of the 484 up-regulated genes. **Table S3.** Transcriptional profiling data for several SA and IAA pathway genes. **Table S4.** Primers used in map-based cloning. **Table S5.** Primers used in the vector construction and mutant analysis. (XLSX 7942 kb)

## Abbreviations

CAPS: Cleaved amplified polymorphic sequence
FA: Fatty acid
FAB2: FATTY ACID BIOSYNTHESIS 2
NPR1: NON-EXPRESSION OF PATHOGENESIS-RELATED GENES 1
PAMP: Pathogen-associated molecular patterns
PDF1.2: PLANT DEFENSIN 1.2
PR1: PATHOGENESIS-RELATED 1
qRT-PCR: Quantitative reverse transcription-PCR
SA: Salicylic acid
SAR: Systemic acquired resistance
SSI2: SUPPRESSOR OF SA INSENSITIVITY 2
SSLP: Simple sequence length polymorphism

## References

[1] Kachroo A, Kachroo P (2009). Fatty acid-derived signals in plant defense. Annu Rev Phytopathol.

[2] Savchenko T, Walley JW, Chehab EW, Xiao Y, Kaspi R (2010). Arachidonic acid: an evolutionarily conserved signaling molecule modulates plant stress signaling networks. Plant Cell.

[3] Routaboul JM, Fischer SF (2000). Trienoic fatty acids are required to maintain chloroplast function at low temperatures. Plant Physiol.

[4] Iba K (2002). Acclimative response to temperature stress in higher plants: approaches of gene engineering for temperature tolerance. Annu Rev Plant Biol.

[5] Yamauchi Y, Furutera A, Seki K, Toyoda Y, Tanaka K (2008). Malondialdehyde generated from peroxidized linolenic acid causes protein modification in heat-stressed plants. Plant Physiol Biochem.

[6] Yu K, Soares JM, Mandal MK, Wang C, Chanda B (2013). A feedback regulatory loop between G3P and lipid transfer proteins DIR1 and AZI1 mediates azelaic-acid-induced systemic immunity. Cell Rep.

[7] Shanklin J, Cahoon EB (1998). Desaturation and related modifications of fatty acids 1. Annu Rev Plant Physiol.

[8] Kachroo A, Shanklin J, Whittle E, Lapchyk L, Hildebrand D (2007). The *Arabidopsis* stearoyl-acyl carrier protein-desaturase family and the contribution of leaf isoforms to oleic acid synthesis. Plant Mol Biol.

[9] Chandra-Shekara AC, Venugopal SC, Barman SR, Kachroo A, Kachroo P (2007). Plastidial fatty acid levels regulate resistance gene-dependent defense signaling in *Arabidopsis*. Proc Natl Acad Sci U S A.

[10] Kachroo P, Shanklin J, Shah J, Whittle EJ, Klessig DF (2001). A fatty acid desaturase modulates the activation of defense signaling pathways in plants. Proc Natl Acad Sci U S A.

[11] Kachroo P, Kachroo A, Lapchyk L, Hildebrand D, Klessig DF (2003). Restoration of defective cross talk in *ssi2* mutants: role of salicylic acid, jasmonic acid, and fatty acids in SSI2-mediated signaling. Mol Plant-Microbe Interact.

[12] Kachroo P, Venugopal SC, Navarre DA, Lapchyk L, Kachroo A (2005). Role of salicylic acid and fatty acid desaturation pathways in ssi2-mediated signaling. Plant Physiol.

[13] Kachroo A, Lapchyk L, Fukushige H, Hildebrand D, Klessig D (2003). Plastidial fatty acid signaling modulates salicylic acid-and jasmonic acid-mediated defense pathways in the *Arabidopsis ssi2* mutant. Plant Cell.

[14] Kachroo A, Venugopal SC, Lapchyk L, Falcone D, Hildebrand D (2004). Oleic acid levels regulated by glycerolipid metabolism modulate defense gene expression in *Arabidopsis*. Proc Natl Acad Sci U S A.

[15] Xia Y, Gao QM, Yu K, Lapchyk L, Navarre D (2009). An intact cuticle in distal tissues is essential for the induction of systemic acquired resistance in plants. Cell Host Microbe.

[16] Venugopal SC, Jeong RD, Mandal MK, Zhu S, Chandra-Shekara AC (2009). Enhanced disease susceptibility 1 and salicylic acid act redundantly to regulate resistance gene-mediated signaling. PLoS Genet.

[17] Sekine KT, Nandi A, Ishihara T, Hase S, Ikegami M (2004). Enhanced resistance to Cucumber mosaic virus in the Arabidopsis thaliana ssi2 mutant is mediated via an SA-independent mechanism. Mol Plant-Microbe Interact.

[18] Mandal MK, Chandra-Shekara AC, Jeong RD, Yu K, Zhu S (2012). Oleic acid-dependent modulation of NITRIC OXIDE ASSOCIATED1 protein levels regulates nitric oxide-mediated defense signaling in *Arabidopsis*. Plant Cell.

[19] Jiang CJ, Shimono M, Maeda S, Inoue H, Mori M (2009). Suppression of the rice fatty-acid desaturase gene OsSSI2 enhances resistance to blast and leaf blight diseases in rice. Mol Plant-Microbe Interact.

[20] Kachroo A, Fu DQ, Havens W, Navarre D, Kachroo P (2008). An oleic acid-mediated pathway induces constitutive defense signaling and enhanced resistance to multiple pathogens in soybean. Mol Plant-Microbe Interact.

[21] Song N, Hu Z, Li Y, Li C, Peng F (2013). Overexpression of a wheat stearoyl-ACP desaturase (SACPD) gene *TaSSI2* in Arabidopsis *ssi2* mutant compromise its resistance to powdery mildew. Gene.

[22] Lightner J, James DW, Dooner HK, Browse J (1994). Altered body morphology is caused by increased stearate levels in a mutant of *Arabidopsis*. Plant J.

[23] Lightner J, Wu J (1994). A mutant of *Arabidopsis* with increased levels of stearic acid. Plant Physiol.

[24] Walters D, Heil M (2007). Costs and trade-offs associated with induced resistance. Physiol Mol Plant Pathol.

[25] Lorrain S, Vailleau F, Balagué C, Roby D (2003). Lesion mimic mutants: keys for deciphering cell death and defense pathways in plants?. Trends in Plant Sci.

[26] Shah J, Kachroo P, Nandi A, Klessig DF (2001). A recessive mutation in the Arabidopsis SSI2 gene confers SA- and NPR1-independent expression of PR genes and resistance against bacterial and oomycete pathogens. Plant J.

[27] Kinkema M, Fan W, Dong X (2000). Nuclear localization of NPR1 is required for activation of PR gene expression. Plant Cell.

[28] Spoel SH, Koornneef A, Claessens SMC, Korzelius JP, Van Pelt JA (2003). NPR1 modulates cross-talk between salicylate-and jasmonate-dependent defense pathways through a novel function in the cytosol. Plant Cell.

[29] Mi H, Muruganujan A, Thomas PD (2013). PANTHER in 2013: modeling the evolution of gene function, and other gene attributes, in the context of phylogenetic trees. Nucl Acids Res.

[30] Du Z, Zhou X, Ling Y, Zhang ZH, Su Z (2010). agriGO: a GO analysis toolkit for the agricultural community. Nucl. Acids Res.

[31] Spartz AK, Lee SH, Wenger JP, Gonzalez N, Itoh H (2012). The SAUR19 subfamily of SMALL AUXIN UP RNA genes promote cell expansion. Plant J.

[32] Chae K, Isaacs CG, Reeves PH, Maloney GS, Muday GK (2012). Arabidopsis SMALL AUXIN UP RNA63 promotes hypocotyl and stamen filament elongation. Plant J.

[33] Lindqvist Y, Huang W, Schneider G, Shanklin J (1996). Crystal structure of delta9 stearoyl-acyl carrier protein desaturase from castor seed and its relationship to other di-iron proteins. EMBO J.

[34] Moche M, Shanklin J, Ghoshal A, Lindqvist Y (2003). Azide and acetate complexes plus two iron depleted crystal structures of the di-iron enzyme Δ9 stearoyl-acyl carrier protein desaturase implications for oxygen activation and catalytic intermediates. J Biol Chem.

[35] Lenardon MD, Munro CA, Gow NA (2010). Chitin synthesis and fungal pathogenesis. Curr Opin Microbiol.

[36] Wang D, Pajerowska-Mukhtar K, Culler AH, Dong X (2007). Salicylic acid inhibits pathogen growth in plants through repression of the auxin signaling pathway. Curr Biol.

[37] Scheideler M, Schlaich NL, Fellenberg K, Beissbarth T, Hauser NC (2002). Monitoring the switch from housekeeping to pathogen defense metabolism in Arabidopsis thaliana using cDNA arrays. J Biol Chem.

[38] Chen S, Songkumarn P, Liu J, Wang GL (2009). A versatile zero background T-vector system for gene cloning and functional genomics. Plant Physiol.

[39] Clough SJ, Bent AF (1998). Floral dip: a simplified method for Agrobacterium-mediated transformation of *Arabidopsis thaliana*. Plant J.

[40] Li N, Kong LG, Zhou WH, Zhang X, Wei ST (2013). Overexpression of *Os2H16* enhances resistance to phytopathogens and tolerance to drought stress in rice. Plant Cell Tiss Org Cult.

[41] Shi H, Ye T, Chen F, Cheng Z, Wang Y (2013). Manipulation of arginase expression modulates abiotic stress tolerance in Arabidopsis: effect on arginine metabolism and ROS accumulation. J Exp Bot.

[42] Gao QM, Venugopal S, Navarre D, Kachroo A (2011). Low oleic acid-derived repression of jasmonic acid-inducible defense responses requires the WRKY50 and WRKY51 proteins. Plant Physiol.

[43] Golani Y, Kaye Y, Gilhar O, Ercetin M, Gillaspy G (2013). Inositol polyphosphate phosphatidylinositol 5-phosphatase9 (At5PTase9) controls plant salt tolerance by regulating endocytosis. Mol Plant.

[44] Brader G, Djamei A, Teige M, Palva ET, Hirt H (2007). The MAP kinase kinase MKK2 affects disease resistance in Arabidopsis. Mol Plant-Microbe Interact.

[45] Mortazavi A, Williams BA, McCue K, Schaeffer L, Wold B (2008). Mapping and quantifying mammalian transcriptomes by RNA-Seq. Nat Methods.

