# Supplementary material for: A novel mutant allele of SSI2 confers a better balance between disease resistance and plant growth inhibition on Arabidopsis thaliana
Source: BMC Plant Biol. 2016 Sep 26;16:208. doi: 10.1186/s12870-016-0898-x (PMC5037883; doi:10.1186/s12870-016-0898-x)
Supplement: Additional file 1: Figure S1. — Mutant test and transgenic plant confirmation. Figure S2. Cytological staining of Col-0 and different ssi2 mutant lines. Figure S3. Quantitative RT-PCR analysis of 14 auxin related genes. Figure S4. Subcellular localization of SSI2 and mutant fragments. (PPTX 1923 kb) [file 12870_2016_898_MOESM1_ESM.pptx]

## Slide 1
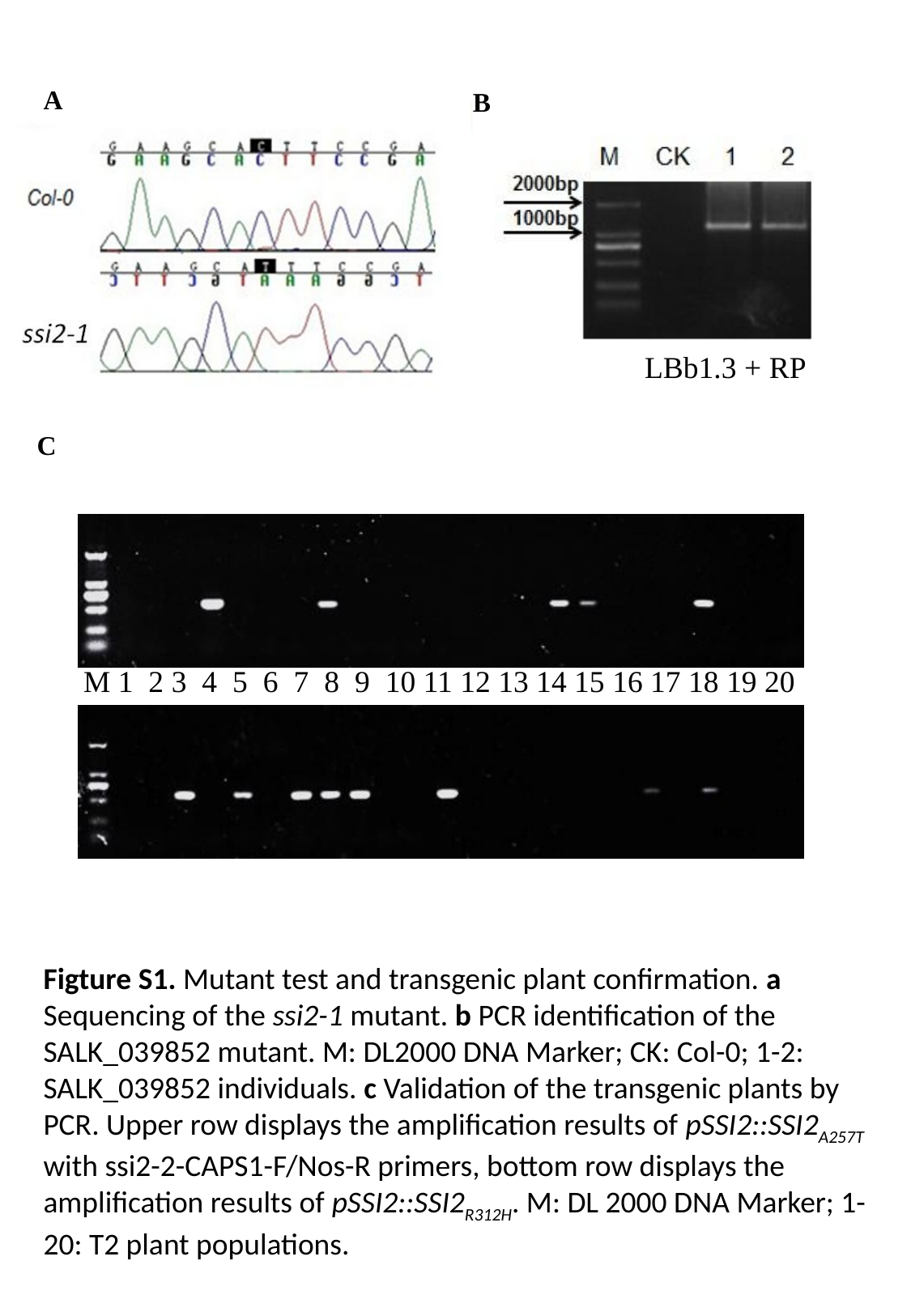

A
B
 LBb1.3 + RP
C
M 1 2 3 4 5 6 7 8 9 10 11 12 13 14 15 16 17 18 19 20
Figture S1. Mutant test and transgenic plant confirmation. a Sequencing of the ssi2-1 mutant. b PCR identification of the SALK_039852 mutant. M: DL2000 DNA Marker; CK: Col-0; 1-2: SALK_039852 individuals. c Validation of the transgenic plants by PCR. Upper row displays the amplification results of pSSI2::SSI2A257T with ssi2-2-CAPS1-F/Nos-R primers, bottom row displays the amplification results of pSSI2::SSI2R312H. M: DL 2000 DNA Marker; 1-20: T2 plant populations.

## Slide 2
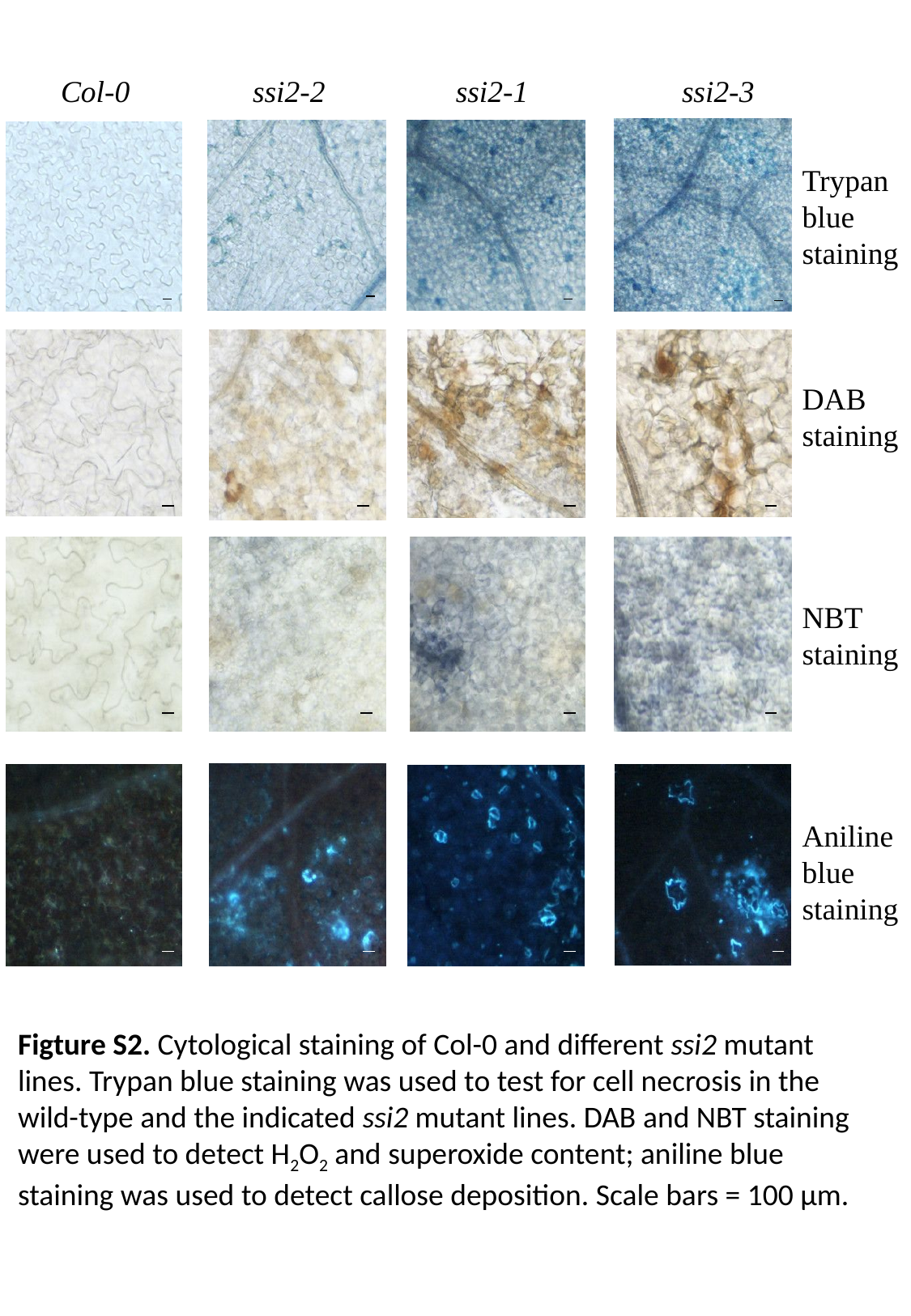

Trypan blue staining
DAB staining
NBT staining
Aniline blue staining
 Col-0 ssi2-2 ssi2-1 ssi2-3
Figture S2. Cytological staining of Col-0 and different ssi2 mutant lines. Trypan blue staining was used to test for cell necrosis in the wild-type and the indicated ssi2 mutant lines. DAB and NBT staining were used to detect H2O2 and superoxide content; aniline blue staining was used to detect callose deposition. Scale bars = 100 μm.

## Slide 3
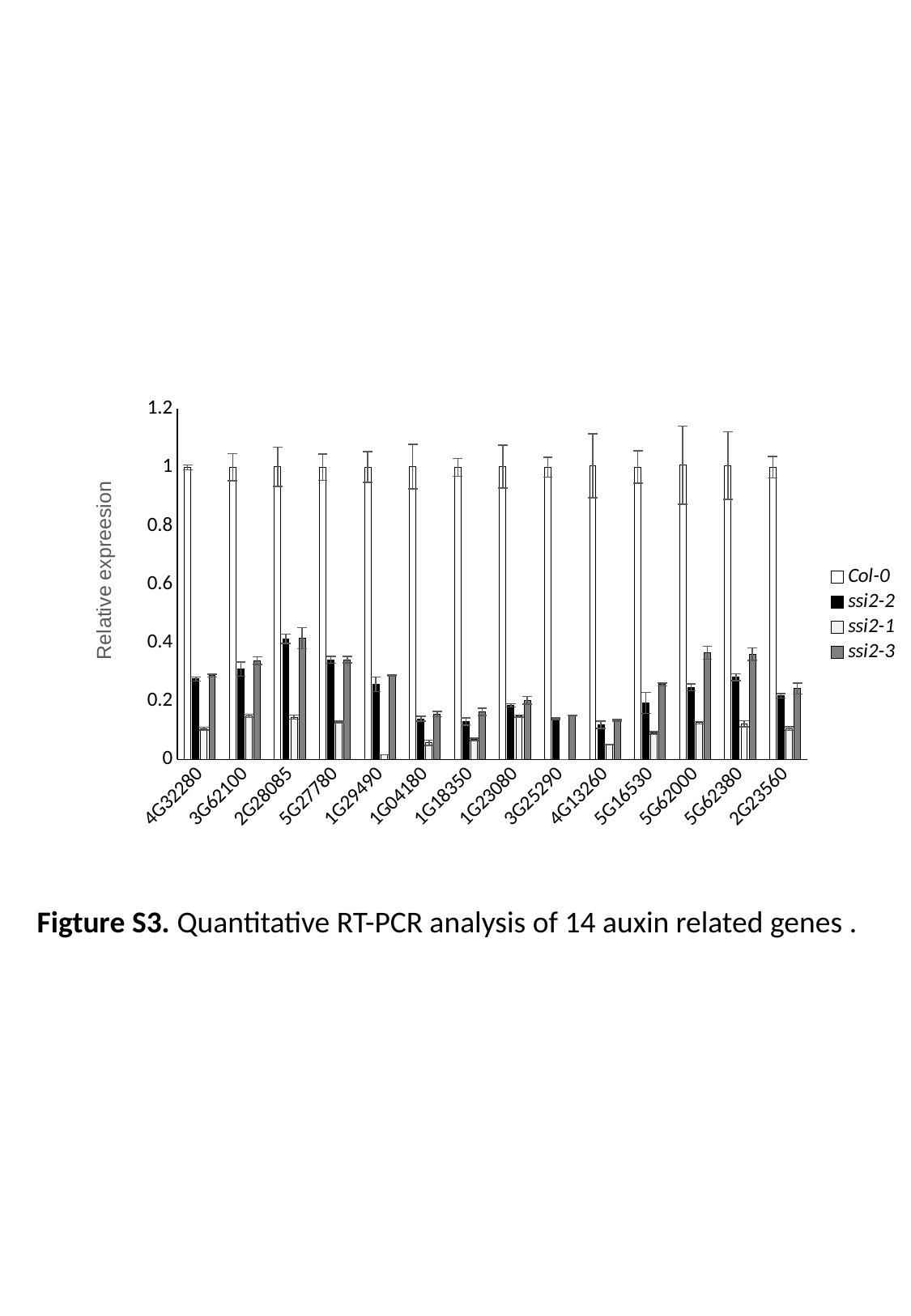

### Chart
| Category | Col-0 | ssi2-2 | ssi2-1 | ssi2-3 |
|---|---|---|---|---|
| 4G32280 | 1.0000330997061209 | 0.2752950782600272 | 0.10567136574229284 | 0.2867549876693964 |
| 3G62100 | 1.0010515017985568 | 0.30898589405357263 | 0.14901170231524788 | 0.33886180824877526 |
| 2G28085 | 1.0022871146482635 | 0.41322293281648653 | 0.14466883013365184 | 0.41514929792286376 |
| 5G27780 | 1.0009954085781876 | 0.34100767202694504 | 0.12895823741964554 | 0.34152196580530614 |
| 1G29490 | 1.0014041035862489 | 0.2574822043269144 | 0.016179492082954047 | 0.28730955858031054 |
| 1G04180 | 1.003020378305805 | 0.13886880114741226 | 0.056733629118856044 | 0.15580380804992652 |
| 1G18350 | 1.0004491413621677 | 0.1303633353997735 | 0.06874835234069882 | 0.16310929735164598 |
| 1G23080 | 1.0026958401841979 | 0.1852102203005307 | 0.14831365196244353 | 0.20221236713883903 |
| 3G25290 | 1.0005839186141035 | 0.13901377913395563 | 1.785790672465815e-05 | 0.15045148433370473 |
| 4G13260 | 1.0057785299114224 | 0.11861539294291869 | 0.05076533949691268 | 0.1341232227099227 |
| 5G16530 | 1.0015573476707476 | 0.19297960477478504 | 0.09154018097814158 | 0.2560688177300621 |
| 5G62000 | 1.0083692626091423 | 0.24707523836651454 | 0.12556736698073845 | 0.3651912950864829 |
| 5G62380 | 1.0070248476513364 | 0.28140319404582365 | 0.1220322415827213 | 0.3605341725762086 |
| 2G23560 | 1.000675897710589 | 0.21806572608624467 | 0.10575784368268575 | 0.24240363803661844 |Figture S3. Quantitative RT-PCR analysis of 14 auxin related genes .

## Slide 4
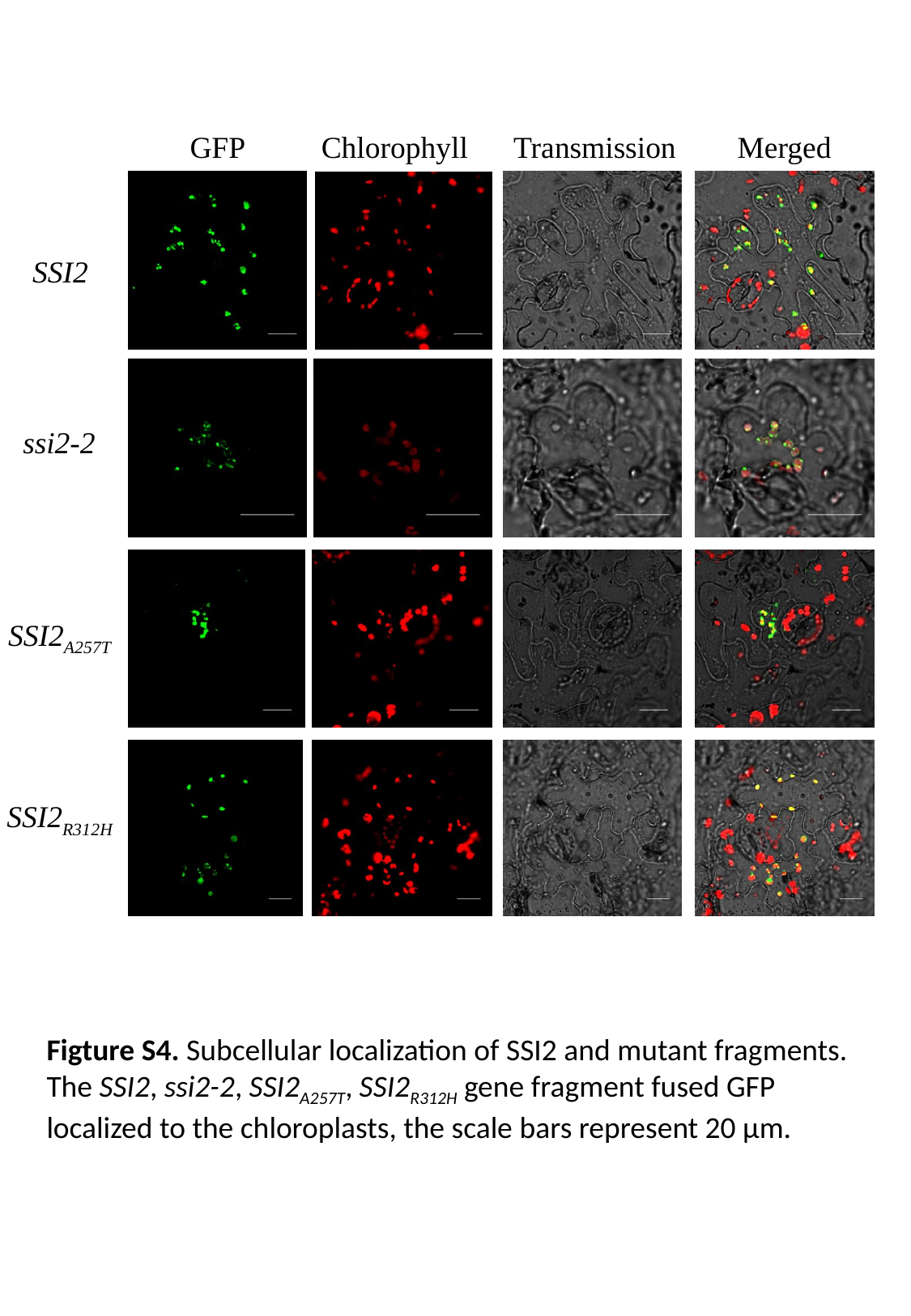

GFP Chlorophyll Transmission Merged
SSI2
ssi2-2
SSI2A257T
SSI2R312H
Figture S4. Subcellular localization of SSI2 and mutant fragments. The SSI2, ssi2-2, SSI2A257T, SSI2R312H gene fragment fused GFP localized to the chloroplasts, the scale bars represent 20 μm.
